# Higher Na^+^-Ca^2+^ Exchanger Function and Triggered Activity Contribute to Male Predisposition to Atrial Fibrillation

**DOI:** 10.3390/ijms231810724

**Published:** 2022-09-14

**Authors:** Simon Thibault, Valérie Long, Céline Fiset

**Affiliations:** 1 Research Center, Montreal Heart Institute, Montréal, QC H1T 1C8, Canada; 2 Faculty of Pharmacy, Université de Montréal, Montréal, QC H3T 1J4, Canada

**Keywords:** atrial fibrillation, sex differences, Na^+^-Ca^2+^ exchanger, Ca^2+^ transient, L-type Ca^2+^ current

## Abstract

Male sex is one of the most important risk factors of atrial fibrillation (AF), with the incidence in men being almost double that in women. However, the reasons for this sex difference are unknown. Accordingly, in this study, we sought to determine whether there are sex differences in intracellular Ca^2+^ homeostasis in mouse atrial myocytes that might help explain male predisposition to AF. AF susceptibility was assessed in male (M) and female (F) mice (4–5 months old) using programmed electrical stimulation (EPS) protocols. Males were 50% more likely to develop AF. The Ca^2+^ transient amplitude was 28% higher in male atrial myocytes. Spontaneous systolic and diastolic Ca^2+^ releases, which are known sources of triggered activity, were significantly more frequent in males than females. The time to 90% decay of Ca^2+^ transient was faster in males. Males had 54% higher Na^+^-Ca^2+^ exchanger (NCX1) current density, and its expression was also more abundant. L-type Ca^2+^ current (I_CaL_) was recorded with and without BAPTA, a Ca^2+^ chelator. I_CaL_ density was lower in males only in the absence of BAPTA, suggesting stronger Ca^2+^-dependent inactivation in males. Ca_V_1.2 expression was similar between sexes. This study reports major sex differences in Ca^2+^ homeostasis in mouse atria, with larger Ca^2+^ transients and enhanced NCX1 function and expression in males resulting in more spontaneous Ca^2+^ releases. These sex differences may contribute to male susceptibility to AF by promoting triggered activity.

## 1. Introduction

Atrial fibrillation (AF) is the most common sustained cardiac arrhythmia. The prevalence of AF is approximately 1–2% in the general population and increases to nearly 10% in elderly individuals [1,2]. These numbers are expected to increase with the aging of the population [3,4]. AF is associated with higher morbidity and mortality and constitutes a major socioeconomic burden [2,5,6]. The most common consequences of AF are stroke and thromboembolic complications. Indeed, AF increases stroke risk 5-fold and is responsible for 25–30% of all ischemic strokes [7,8,9].

Together with ageing and genetic predispositions, male sex is one of the major risk factors of AF [2,10,11,12]. In fact, after adjusting for age and other predisposing conditions, the risk of developing AF is 1.5 to 2 times higher in men compared to women [1,12,13,14,15]. Moreover, not only do men have a higher prevalence of AF, but they are diagnosed at a younger age than women [13]. Currently, pharmacological treatments for AF have limited efficacy, and adverse effects such as proarrhythmic events can often be encountered [16,17,18]. It is therefore essential to find more effective and safer therapeutic strategies. For these reasons, over the past decades, considerable energy has been devoted to understanding the mechanisms of AF, but the differences between men and women with AF pathophysiology have received little attention [13]. However, to develop an improved therapeutic approach, there is a critical need for a detailed understanding of the cellular and molecular mechanisms underlying AF sex-related differences.

AF is associated with substantial changes in atrial structural, electrophysiological, and intracellular Ca^2+^ properties. The remodeling of the atria is a critical process involved in the initiation and maintenance of AF [16,17,18]. While the maintenance of AF is strongly dependent on electrical and structural remodeling, alterations in intracellular Ca^2+^ homeostasis play a major role in ectopic-triggered activity involved in AF initiation. During the action potential, extracellular Ca^2+^ enters the cell via L-type Ca^2+^ channels (Ca_V_1.2), triggering sarcoplasmic reticulum (SR) Ca^2+^ release through SR-bound ryanodine receptors (RyR2). This Ca^2+^-induced Ca^2+^ release leads to a large and transient rise in intracellular Ca^2+^, which drives myocyte contraction. Then, the concerted action of SR Ca^2+^-ATPase (SERCA2a) and sarcolemmal Na^+^-Ca^2+^ exchanger (NCX1) reduces cytoplasmic Ca^2+^ to basal levels [19]. Disturbances in these Ca^2+^ cycling processes, such as elevated intracellular Ca^2+^, are known to cause spontaneous Ca^2+^ releases, which can lead to delayed afterdepolarizations (DAD), focal ectopic-triggered activity, and initiation of AF [16].

Accordingly, the main objectives of this study were to first determine whether the male prevalence of AF occurring in humans was also present in mice, and to determine whether there are differences in atrial Ca^2+^ homeostasis between healthy male and female mice that may contribute to the increased risk of AF in males, without the effect of aging or other AF risk factors.

## 2. Results

### 2.1. Increased Susceptibility to AF in Male Mice

**Figure 1:**
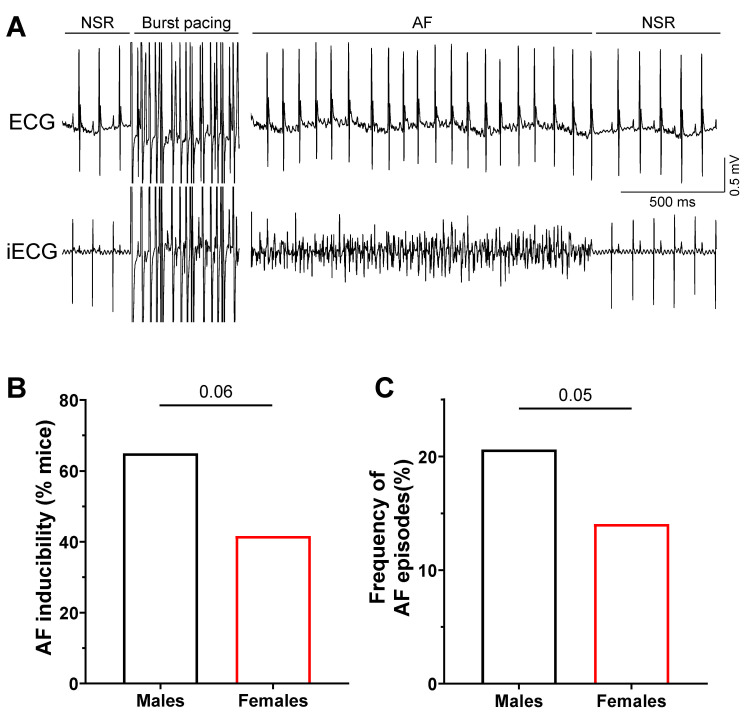
**Higher susceptibility to AF in males in response to EPS.** (**A**) Typical recording of a lead I surface ECG with simultaneous intracardiac ECG (iECG) during a sequence of normal sinus rhythm (NSR), burst pacing, sustained atrial fibrillation (AF), and recovery to normal sinus rhythm. The burst pacing was cropped lengthwise for the purpose of this example. (**B**) Males developed more AF in response to the burst pacing protocol, presented as the percentage of mice who developed at least one sustained AF (M: 65%, 13/20; F: 42%, 10/24; *p* = 0.06, one-sided chi-squared test). (**C**) Frequency of AF episodes, as the percentage of AF episodes induced out of the total number of simulations (8 per animal), was higher in males (M: 20%, 33/160; F: 14%, 27/192; *p* = 0.05, one-sided chi-square test).

Spontaneous AF episodes are unlikely in these healthy adult mice and indeed were not observed in electrocardiogram (ECG) recordings obtained prior to stimulation protocols. Therefore, a burst stimulation protocol designed to elicit AF was used to examine the susceptibility to AF of male and female mice. Data presented in Figure 1 illustrate an example of inducible AF in a male mouse obtained using electrophysiological programmed stimulation (EPS) protocols and show that males developed AF more frequently than females in response to burst pacing. Specifically, 65% (13/20) of males developed at least one episode of AF, compared to only 42% (10/24, *p* = 0.06) of females (Figure 1B). The EPS protocol was repeated 8 times per mouse and AF episodes were more frequent in males (21%, 33 AF/160 stimulations) than in females (14%, 27 AF/192 stimulations, *p* = 0.05) (Figure 1C). For both parameters, males were 50% more likely than females to develop AF in response to stimulation. Taken together, these results suggest that, like humans, male mice are more vulnerable to AF than females.

### 2.2. Ca^2+^ Transient Amplitude Is Larger in Male Than Female Atrial Myocytes

**Figure 2:**
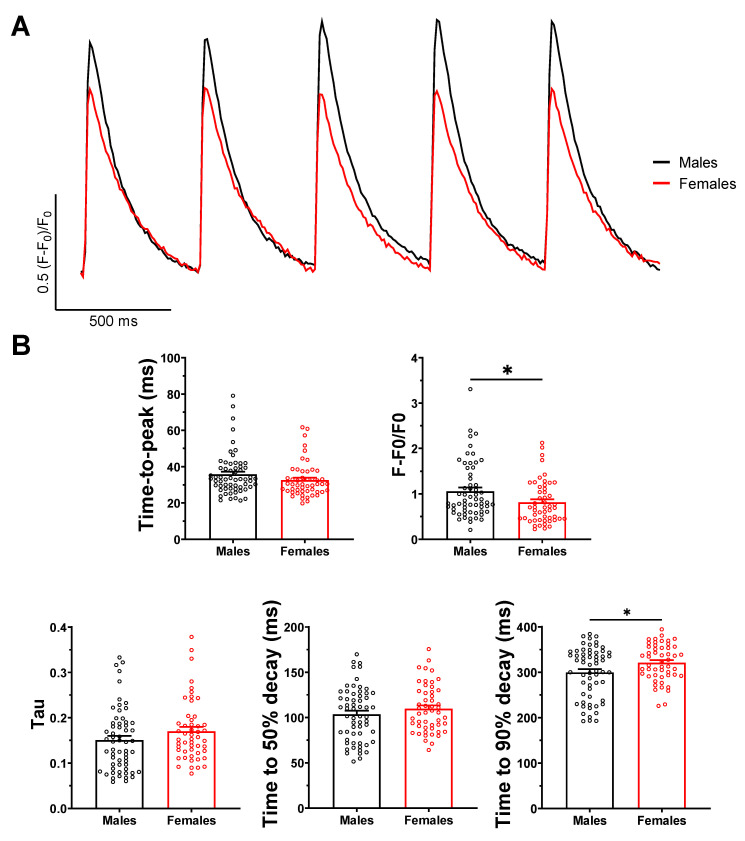
**Ca^2+^ transients from male atrial myocytes have greater amplitude and faster decay than females.** (**A**) Typical Ca^2+^ transients of male (black) and female (red) atrial myocytes, under 2 Hz field stimulation, generated using Fluo-4-AM Ca^2+^-sensitive dye in confocal microscopy. (**B**) Ca^2+^ transients of male myocytes have a greater amplitude (F-F_0_/F_0_) than female (M: 1.06 ± 0.08; F: 0.81 ± 0.07; * *p* = 0.02). Time-to-peak, tau of decay, and time to 50% decay were not statistically different between sexes. Time to 90% decay was faster in males (M: 300 ± 7 ms; F: 321 ± 6 ms; * *p* = 0.02) (for each parameter: M: n = 59–60, N = 3; F: n = 50–51, N = 3). * *p* < 0.05

Intracellular Ca^2+^ is critical for efficient atrial function, and remodeled Ca^2+^ handling is known to play an important role in the initiation of AF [20,21,22]. Therefore, we sought to determine whether changes in Ca^2+^ regulation in males could be associated with their higher AF susceptibility. Data presented in Figure 2 show Ca^2+^ transient recordings of left atrial myocytes obtained from male and female mice. Myocytes from both sexes displayed similar diastolic fluorescence levels (F_0_). However, the amplitude of the Ca^2+^ transients was 30% larger in males than in females (M: 1.06 ± 0.08, n = 59, N = 3; F: 0.81 ± 0.07, n = 51, N = 3; *p* = 0.02) (Figure 2B). In addition, the 90% decay of the Ca^2+^ transient was faster in males (300 ± 7 ms, n = 60) than in females (321 ± 6 ms, n = 51; *p* = 0.02).

### 2.3. The Frequency of Spontaneous Ca^2+^ Release Is Higher in Male Mice

**Figure 3:**
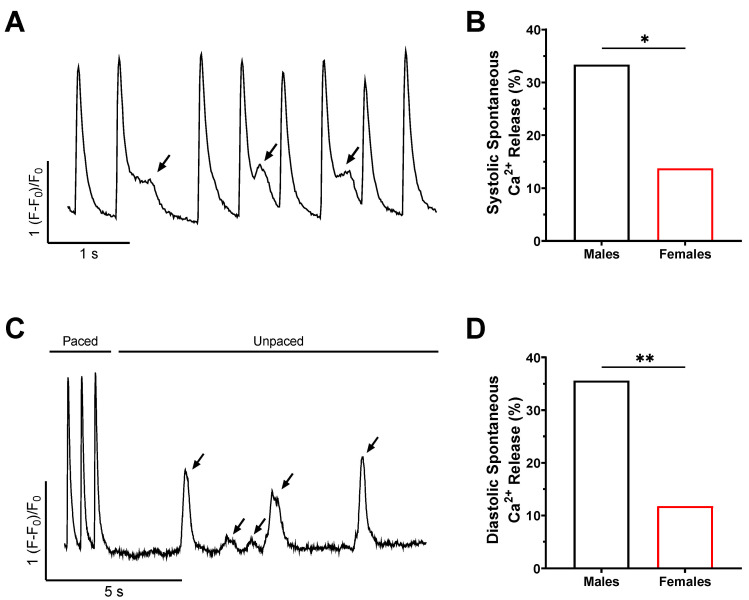
**Spontaneous Ca^2+^ releases are more frequent in male atrial myocytes.** (**A**) Typical example of a Ca^2+^ transient recording on a freshly isolated atrial myocyte exhibiting systolic spontaneous Ca^2+^ releases during the 10 s of 2 Hz pacing. Events are highlighted by the arrows. (**B**) Percentage of cells in which systolic spontaneous Ca^2+^ releases were observed (M: 33%, 20/60, N = 3; F: 14%, 7/51, N = 3; * *p* = 0.02, two-sided chi-squared test). (**C**) Typical example of a recording showing spontaneous Ca^2+^ releases during diastole, during the 10 s without pacing. Events are highlighted by the arrows. (**D**) Percentage of cells in which diastolic spontaneous Ca^2+^ releases were observed (M: 36%, 21/59, N = 3; F: 12%, 6/51, N = 3; ** *p* = 0.004, two-sided chi-squared test).

When acquiring and analyzing Ca^2+^ transient data, we observed several spontaneous Ca^2+^ releases (Figure 3). These events occurred both during stimulation and at rest and were identified as spontaneous systolic and diastolic Ca^2+^ releases, respectively. Figure 3 illustrates representative spontaneous systolic and diastolic Ca^2+^ release recorded following stimulated Ca^2+^ transients as well as the quantification of these events. The proportion of cells that displayed spontaneous systolic and diastolic Ca^2+^ activity was much larger in males (systolic, 33%, n = 60; diastolic: 36%, n = 59) compared to females (systolic, 14%, n = 51, *p* = 0.02; diastolic: 12%, n = 51, *p* = 0.004). Altogether, these results strongly suggest that these sex differences in intracellular Ca^2+^ handling may be central to the male susceptibility to AF, as Ca^2+^ leak is known to drive triggered activity.

### 2.4. Enhanced NCX1 Function and Expression in Male Mice

**Figure 4:**
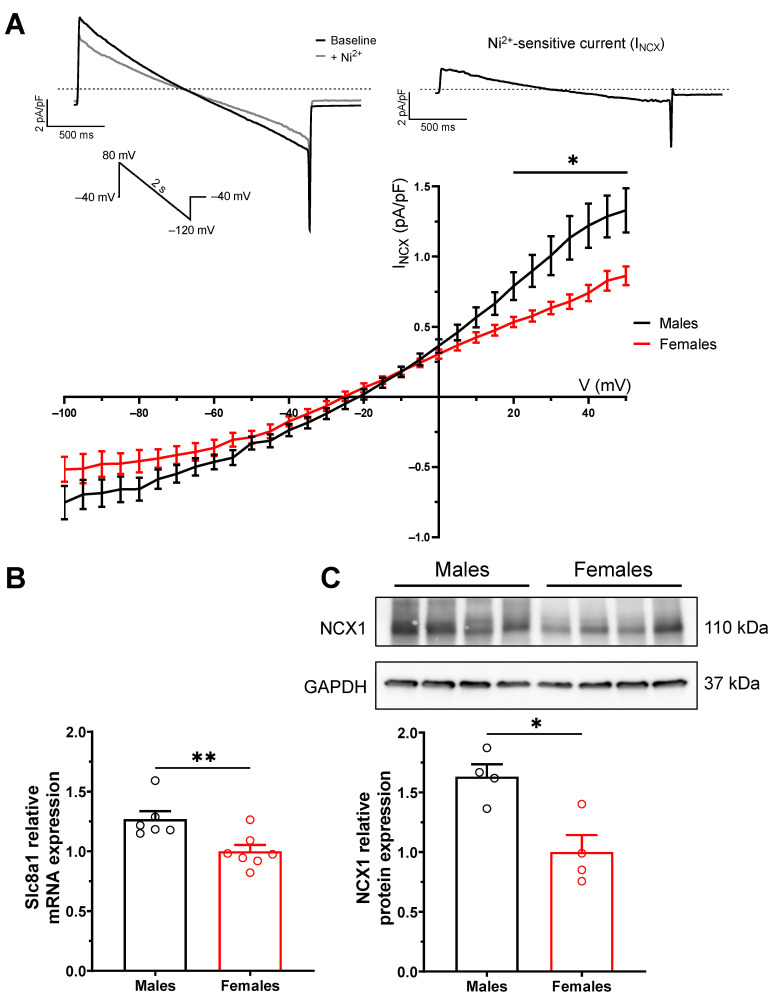
**Higher I_NCX_ and NCX1 expression in male atria than in females.** (**A**) Top left: Typical recording in baseline conditions (black) and following inhibition of NCX1 with 5 mM NiCl (grey). Voltage protocol is shown in inset. Top right: Typical example of the nickel-sensitive subtracted I_NCX_. Dotted line represents the zero-current. Bottom: Mean IV curves of I_NCX_ show larger current density in males than females (* *p* ˂ 0.05 from +20 to +50 mV; M: n = 18–19, N = 3; F: n = 13, N = 3). (**B**) Scatter plot shows a higher mRNA expression of *Slc8a1* (coding for NCX1) in males than in females (M: 1.27 ± 0.07, n = 6; F: 1.00 ± 0.05, n = 7; ** *p* = 0.009). (**C**) Western blot of NCX1 in male and female left atria and their respective loading control GAPDH. Densitometry analysis revealed a higher expression of NCX1 in males (M: 1.63 ± 0.10, n = 4; F: 1.00 ± 0.14, n = 4; 2 left atria/n; * *p* = 0.01). * *p* < 0.05, ** *p* < 0.01

The role of NCX1 in the regulation of the intracellular Ca^2+^ concentration was then explored in atrial myocytes of male and female mice. NCX1 was of particular interest as it is known to play a major role in triggered activity involved in AF initiation [20,21,22,23,24]. Accordingly, I_NCX_ measured as Nickel-sensitive current was compared between male and female atrial myocytes. Results reported in Figure 4 show that the density of I_NCX_ was 54% larger in males (at +50 mV: 1.33 ± 0.16 pA/pF, n = 19, N = 3) compared to females (0.86 ± 0.07 pA/pF, n = 13, N = 3; *p* = 0.002). Consistently, NCX1 mRNA and protein expression was significantly higher in atrial tissues of males than females, as assessed by quantitative polymerase chain reaction (qPCR) (27%, *p* = 0.009) and Western blot analysis (63%, *p* = 0.01).

### 2.5. Sex Difference in L-Type Ca^2+^ Current (I_CaL_) Is Due to Difference in Intracellular Calcium

**Figure 5:**
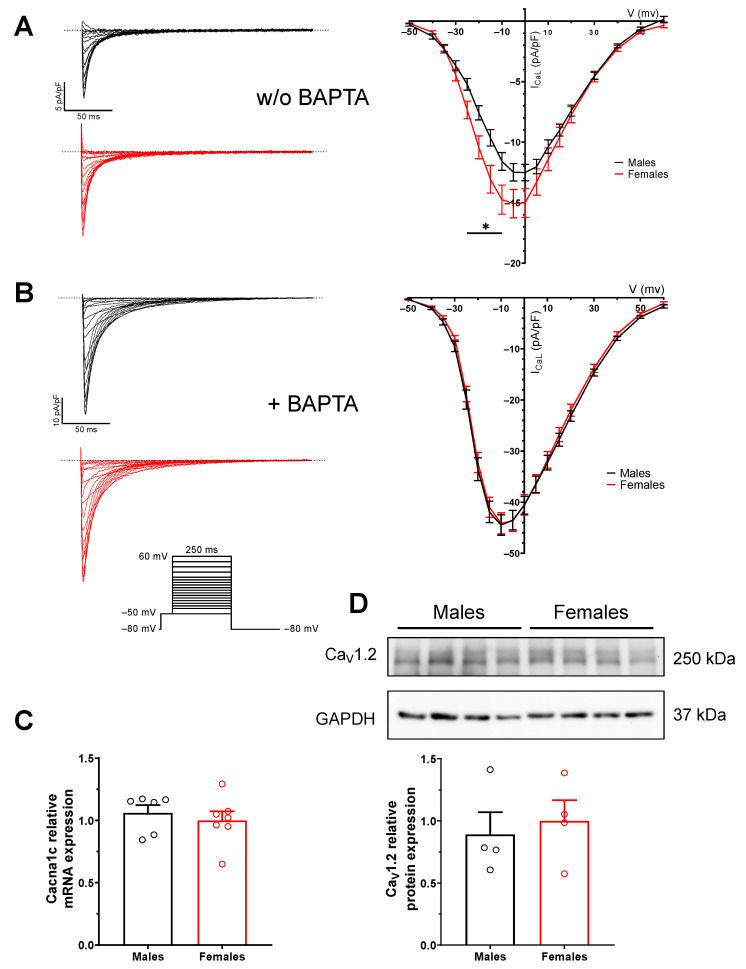
**Similar I_CaL_ density and Ca_V_1.2 expression in male and female mouse atria.** (**A**) Left: Typical recordings of I_CaL_ from male (black) and female (red) atrial myocytes in the absence of BAPTA (inset displays the voltage protocol). Dotted line represents the zero current. Right: Mean IV curves of I_CaL_ show a lower current density in males (* *p* ˂ 0.05 from –25 to −10 mV; M: n = 22, N = 3; F: n = 21, N = 2). (**B**) Left: Typical recordings of I_CaL_ from male (black) and female (red) atrial myocytes with 10 µM BAPTA in the pipette solution. Right: Buffering of intracellular Ca^2+^ by BAPTA abolishes the difference in I_CaL_ density between males and females, as shown on mean IV curves (*p* > 0.05 at all voltages; M: n = 26, N = 2; F: n = 25, N = 2). (**C**) The mRNA expression of *Cacna1c* (underlying Ca_V_1.2) is similar in male and female atria (M: 1.06 ± 0.06, n = 6; F: 1.00 ± 0.07, n = 7; *p* = 0.6). (**D**) Densitometry analysis of Western blot of Ca_V_1.2 reveals that protein expression is similar in male and female atria. GAPDH was used as a loading control, for normalization (M: 0.89 ± 0.18, n = 4; F: 1.00 ± 0.17, n = 4; *p* = 0.7).

Next, I_CaL_ was recorded as Ca^2+^ influx through Ca_V_1.2 provides the trigger to initiate the Ca^2+^-induced Ca^2+^-release from the SR, which results in a transient increase in intracellular Ca^2+^ concentrations (Ca^2+^ transients). Ca_V_1.2 is tightly regulated by intracellular Ca^2+^ via calmodulin binding [19]. Therefore, given its strong Ca^2+^-dependent inactivation, I_CaL_ was recorded in the presence and absence of the Ca^2+^ chelator, BAPTA, in the pipette solution (Figure 5). Data reported in Figure 5A show that without BAPTA, I_CaL_ density was 20% lower in males (at −10 mV: −11.6 ± 0.7 pA/pF, n = 22, N = 3) than females (−14.8 ± 1.2 pA/pF, n = 21, N = 2, *p* = 0.03). However, in the presence of BAPTA (Figure 5B), males and females had similar current densities (at −10 mV: M: −44.5 ± 1.9 pA/pF, n = 26, N = 2; F: −44.2 ± 2.1 pA/pF, n = 25, N = 2; *p* = 0.92). Thus, intracellular Ca^2+^ buffering abolished the sex difference in I_CaL_ density. Further analysis of activation and inactivation kinetics properties of I_CaL_ are presented in Supplementary Figures S1 and S2 and Table S3. Specifically, we found a positive shift in the steady-state activation curve of I_CaL_ along with a slower slope in males only in the absence of BAPTA (Figure S1). Importantly, Ca_V_1.2 mRNA and protein expression were similar between the two groups (Figure 5C,D), confirming that the sex difference in I_CaL_ (in the absence of BAPTA) is due to regulation of I_CaL_ by intracellular Ca^2+^ and/or changes in activation kinetics rather than differences in Ca_V_1.2 channel expression.

### 2.6. RyR2 Gene Expression Is Increased in Female Atria 

Gene expression of *Ryr2*, *Atp2a2* (coding SERCA2a), and *Pln* (coding for phospholamban), other important components of intracellular Ca^2+^ homeostasis, was also compared between males and females. The only difference found was for *Ryr2,* which was 18% lower in male mouse atria (Figure S3). Thus, *Ryr2* expression does not account for the larger Ca^2+^ transient amplitudes in males.

### 2.7. Increased NCX1 Gene Expression in Human Atrial Tissues 

**Figure 6:**
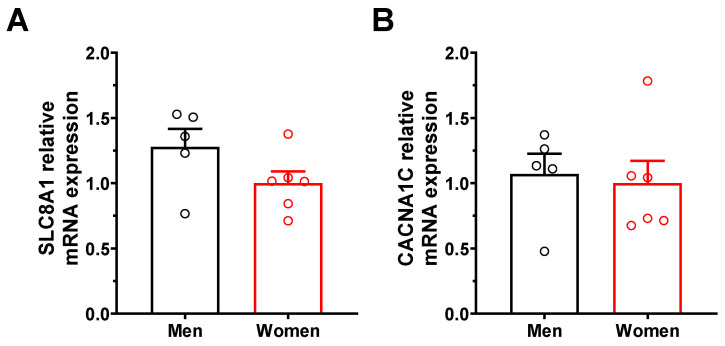
***SLC8A1* and *CACNA1C* gene expression in human atria.** Relative mRNA expression of (**A**) *SLC8A1* (coding for NCX1) and (**B**) *CACNA1C* (coding for Ca_V_1.2) in human atrial tissues from men and women without heart disease. The expression of genes of interest was normalized to the housekeeping gene *HPRT1* (*SLC8A1*: M: 1.28 ± 0.14, n = 5; F: 1.00 ± 0.09, n = 6; *p* = 0.12; *CACNA1C*: M: 1.07 ± 0.16, n = 5; F: 1.00 ± 0.17, n = 6; *p* = 0.77).

We then took advantage of human atrial tissues available through the MHI human tissue bank to explore whether there were differences in NCX1 and Ca_V_1.2 expression in atrial tissues of explanted hearts from 5 men and 6 women without heart disease. Consistent with results obtained in mice, qPCR data reported in Figure 6 show similar expression of *CACNA1C* in atrial tissues from men and women, whereas the expression of *SLC8A1* was 28% higher in men than in women. Although not statistically different (*p* = 0.12) due to the limited number of samples available, the difference in human SLC8A1 gene expression suggests that mechanisms similar to those seen in mice might also apply to humans.

### 2.8. NCX1 Gene Expression Is Unchanged in Orchiectomized (ORC) and Ovariectomized (OVX) Mice

**Figure 7:**
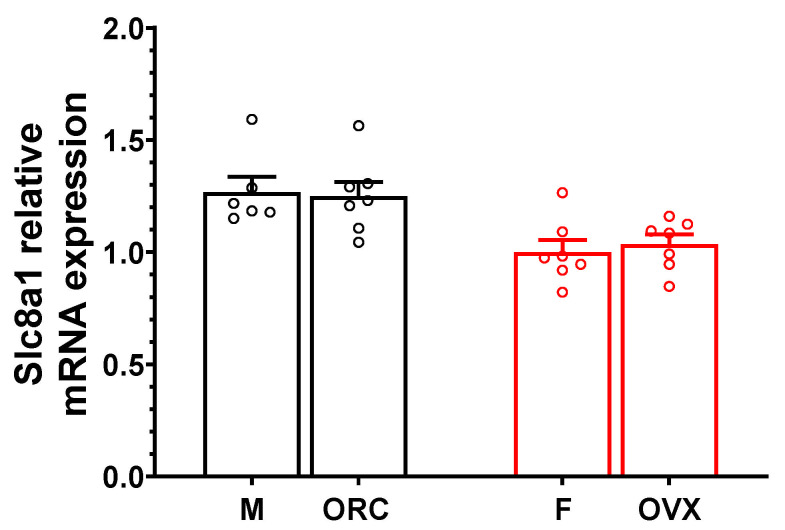
Atrial *Slc8a1* gene expression is not affected by orchiectomy (ORC) and ovariectomy (OVX) in mice. Scatter plot shows no difference in the mRNA expression of *Slc8a1* in ORC and OVX compared to their respective controls (N = 6–7/group).

Lastly, to determine whether male and female sex hormones were involved in the sex difference found in NCX1 mRNA expression, we performed additional qPCR experiments on left atrial tissues from ORC male and OVX female mice. Data reported in Figure 7 show that there was no difference in *Slc8a1* expression in ORC and OVX mice compared to their respective controls, suggesting that sex hormones do not regulate NCX1 expression in the atria. Additionally, *Cacna1c* expression was not affected by either orchiectomy or ovariectomy (Supplemental Figure S4), consistent with data obtained in intact mice.

## 3. Discussion 

Summary of main findings. Clinical observations show that men have a higher incidence and earlier onset of AF compared to women [1,2,12,13,14,15]. However, the mechanisms underlying men’s greater susceptibility to AF remain to be elucidated. In this study, we first reported that the male prevalence of AF observed in humans is also present in mice. Indeed, we found that male mice are 50% more vulnerable to AF than females in response to atrial burst stimulations. Male atrial myocytes have larger Ca^2+^ transient amplitudes with faster decay. They also display higher NCX1 current density and expression as well as reduced I_CaL_ density in males. These sex differences in intracellular Ca^2+^ handling were associated with more frequent spontaneous Ca^2+^ releases in males, suggesting that male atria are more susceptible to triggered activity, which promotes AF initiation.

Alterations in Ca^2+^ handling are among the main causes of AF development. Indeed, AF is often associated with Ca^2+^ overload, which frequently leads to spontaneous Ca^2+^ releases, ectopy, and triggered activity [20,21,22]. In addition, NCX1 overexpression, increased spontaneous Ca^2+^ release, and reduced I_CaL_ have all been associated with AF. One of the main findings of this study is that by using healthy adult male and female mice, we were able to demonstrate that these parameters involved in AF development are already different in atrial myocytes from healthy adult male mice compared to their age-matched female counterparts. These findings reveal important sex differences in intracellular Ca^2+^ regulatory mechanisms that may help explain why male atrial myocytes become more susceptible to developing AF once aging and other AF comorbidities are added to the picture.

Although a number of studies have addressed the influence of sex on intracellular Ca^2+^ homeostasis in ventricular myocytes, very little is known about sex-related differences in intracellular Ca^2+^ regulatory mechanisms in atrial myocytes [25,26,27,28,29]. Nonetheless, several ventricular studies have reported results in good agreement with the results reported here. First, there is clear consensus that Ca^2+^ transients are higher in males than in females [25]. Additionally, similar to our findings, Farell et al. found that the SR Ca^2+^ content was similar in ventricular myocytes from male and female animals [26]. Most importantly, they showed a greater SR Ca^2+^ release in males with no difference in I_CaL_ density, indicating that male ventricular myocytes exhibit a higher gain of EC coupling, which represent the amount of SR Ca^2+^ released per unit of Ca^2+^ current [26]. Interestingly, sex differences in these mechanisms also appear to operate in atrial myocytes, where for equal or less Ca^2+^ influx into the cell, the amplitude of Ca^2+^ transients is greater in males.

To determine whether differences in RyR2 could explain the sex difference in Ca^2+^ transients, we compared *Ryr2* mRNA expression in the atria of male and female mice and found that *Ryr2* was more abundant in females. Interestingly, several reports have found similar results in the ventricles, where RyR2 mRNA and protein levels are significantly higher in females than males [30,31]. Together, these data indicate that an increased RyR2 level does not account for the larger Ca^2+^ transients in male cardiomyocytes. The significance of this is unclear, perhaps it is a compensatory mechanism to attenuate the higher gain of EC coupling in males.

Upregulation of NCX1 expression levels and its corresponding current (I_NCX_) has been consistently reported in AF patients and animal models and is thought to contribute to the generation of delayed afterdepolarizations (DADs) and triggered activity involved in the initiation of AF [16,24]. NCX1 is an electrogenic ion transporter that exchanges three Na^+^ ions for one Ca^2+^ ion in a forward or a reverse mode, depending on the Na^+^ and Ca^2+^ electrochemical gradients. In its forward mode, NCX1 extrudes 1 Ca^2+^ ion in exchange of 3 Na^+^ ions, which generates a depolarizing inward current. DADs are related to abnormal increases in intracellular Ca^2+^ transients, which activate a transient inward current (I_ti_) carried mainly by inward I_NCX_. Accordingly, a larger I_NCX_, as seen in males, will accentuate this phenomenon and facilitate the transition of DADs into spontaneous action potentials. In its reverse mode, NCX1 generates a repolarizing outward current from the influx of 1 Ca^2+^ and efflux of 3 Na^+^ ions. This mode is most active very early into the action potential. Thus, in the reverse mode, the greater density of I_NCX_ in males could participate in the initiation of Ca^2+^-induced Ca^2+^ release and, thus, contribute to the higher Ca^2+^ transient amplitude seen in males. Under physiological conditions, the reverse mode of NCX1 contributes less to Ca^2+^ homeostasis than its forward mode. Nonetheless, the reverse mode of NCX1 is still important in the context of AF. In dogs, blocking the reverse mode of NCX1 with KB-R7943 prevented electrical remodeling caused by rapid atrial pacing and AF [32]. In clinical settings, NCX1 has been consistently reported to be upregulated in AF patients [33,34,35,36]. Consistent with these observations, in this study, we found that NCX1 current density and expression were higher in male mice. Equally important, these changes were associated with increased spontaneous Ca^2+^ release. Together, these sex differences could contribute to the male susceptibility to AF by promoting triggered activity.

I_CaL_ plays a central role in atrial remodeling associated with AF [20,21,22]. Specifically, the onset of AF has been shown to cause Ca^2+^ overload and a reduction in I_CaL_ that leads to progressive shortening of the atrial effective refractory period (AERP), which facilitate the onset of DADs and maintenance of AF. While there is a consensus regarding the reduction of I_CaL_ in response to AF, data on Ca_V_1.2 expression are inconsistent [37,38,39,40,41]. In a recent study on human atrial myocytes from patients without AF or with longstanding AF, I_CaL_ density was shown to be reduced in the presence of AF only in men but not in women [37]. Moreover, this reduction was not due to differences in the expression of Ca_V_1.2 [37]. In the present study, we compared I_CaL_ in male and female mice without AF and found that the density of I_CaL_ was lower in males, although Ca_V_1.2 expression was similar between sexes. Interestingly, buffering intracellular Ca^2+^ with BAPTA abolished the sex difference in I_CaL_ density. Since the Ca^2+^ transient amplitude is 30% higher in males, their I_CaL_ is expected to undergo stronger Ca^2+^-dependent inactivation, to provide a negative feedback mechanism to limit Ca^2+^ influx. Thus, the lower I_CaL_ density in males is likely a consequence of its increased Ca^2+^-dependent inactivation as well as enhanced NCX1 function and is not related to changes in Ca_V_1.2 channel expression. Hence, we found sex-dependent differences in these healthy animals in the mechanisms involved in AF-induced atrial remodeling that may contribute to greater male vulnerability to AF.

It is now becoming clear that sex steroid hormones may be important factors in the development of some types of ventricular arrhythmia (e.g., torsades de pointe), but their role in the male predominance of AF remains to be established [12,42,43,44]. Indeed, the literature on the contribution of sex hormones to AF is very limited and contradictory, with both estrogen and testosterone being associated with an increased risk of AF in some studies and a lower risk in other reports [14]. In this study, to explore the contribution of sex hormones, we used gonadectomized male and female mice of the same age as our healthy control mice. Our qPCR experiments showing that ORC and OVX had no effect on *Slc8a1* expression provide strong evidence that in the atria, NCX1 expression is not regulated by sex hormones and is therefore not involved in the sex differences we observed in NCX1 expression and function. Of note, as shown in Figure S4, *Cacna1c* expression was not different between male and female atrial tissues, nor was it affected by orchiectomy or ovariectomy. Interestingly, NCX1 and Ca_V_1.2 have been reported to be more abundant in female rat ventricles in comparison to males [30,45,46,47]. Moreover, NCX1 and Ca_V_1.2 have been shown to be upregulated by estrogen in rabbit and human ventricles [48]. This apparent discrepancy in the regulation of NCX1 and Ca_V_1.2 by sex hormones between atria and ventricles highlights potential chamber- and/or species-specific differences. Interestingly, with respect to our NCX1 and Ca_V_1.2 expression data, we observed results similar to those obtained in mice using human atrial tissues from men and women without heart disease. Consistent with the murine data, *CANCA1C* expression was found to be similar between atrial tissues from men and women. Interestingly, NCX1 mRNA expression was 28% higher in men than in women, like what we observed in mice. However, due to the limited number of human samples available, this difference, while interesting, was not statistically different, and requires a larger sample size to be validated. Nevertheless, knowing that several reports have shown that NCX1 is upregulated in AF patients, it is tempting to assume that men could have higher NCX1 expression even in the absence of AF, which would help explain their greater vulnerability to AF [33,34,35,36]. Although there are important differences in human and mouse cardiac electrophysiology, the mechanisms of intracellular Ca^2+^ regulation are similar between both species. However, NCX1 has a larger contribution in humans, where it is responsible for almost a third of the Ca^2+^ extrusion, compared to contributing to less than 10% in mice [19]. Thus, sex differences in NCX1 activity and expression would have even greater physiological effects in humans.

## 4. Material and Methods

### 4.1. Animals

The animals used in this study were 4–5-month-old male and female CD-1 mice purchased from Charles River (St-Constant, Qc, Canada). All experiments were conducted in accordance with the Canadian Council on Animal Care (CCAC) and the Guide for the Care and Use of Laboratory Animals published by the National Research Council (NIH Publication No. 85–23, 8th ed. 2011). All experiments were approved by the Montreal Heart Institute Animal Care Committee (2015-80-03, 2018-80-02, and 2021-80-01). A subset of experiments was carried out using ORC male and OVX female mice. Orchiectomy and ovariectomy were performed as previously described, on 30-day-old male mice and 2-month-old female mice [42,43,44,49]. ORC and OVX mice were studied when they reached 4–5 months of age.

### 4.2. Electrophysiological Programmed Stimulation (EPS) Studies

Susceptibility to AF was assessed with EPS, as previously described [50,51]. Briefly, induction of supraventricular arrhythmias was tested using a Transonic (Ithaca, NY, USA) 1.9F octapolar electrophysiology catheter inserted into the right atrium via the right jugular vein of mice, under anesthesia (2% isoflurane). Body temperature was maintained at 37 °C with a heating pad. Bipolar intracardiac ECG (iECG) was obtained from the two most distal pairs of electrodes and lead I surface ECG was simultaneously recorded. Adequate placement of the catheter in the right atrium was achieved when the main deflection of the iECG coincided with the P wave of the surface ECG. Each mouse underwent an identical stimulation protocol (5 s at S1S1: 50–10 ms, 10 ms stepwise reduction). The burst stimulation protocol was performed at twice the threshold intensity and was repeated 8 times on each mouse. AF was defined as an irregular and rapid atrial rhythm with a variable ventricular rate, lasting for at least 1 s and measured from the end of the stimulation until the first P wave of normal sinus rhythm on the ECG.

### 4.3. Isolation of Mouse Atrial Myocytes

Single mouse atrial myocytes were isolated using enzymatic digestion by Langendorff retrograde perfusion, as previously reported [51,52,53]. Left atrium was used as it is more vulnerable to the development of AF [20,54]. Briefly, mice were heparinized (100 USP units IP) 15 min prior to sacrifice to prevent coagulation. The mice were anesthetized by inhalation of isoflurane (2%) and sacrificed by cervical dislocation. The heart was rapidly removed, and retrogradely perfused through the aorta on a modified Langendorff apparatus at a constant flow (2.0 ± 0.1 mL/min) and temperature (37 ± 1 °C). The heart was perfused with the following solutions: (1) 5 min with HEPES-buffered Tyrode’s solution (in mM: 130 NaCl, 5.4 KCl, 1 CaCl_2_, 1 MgCl_2_, 0.33 Na_2_HPO_4_, 10 HEPES, 5.5 glucose, pH adjusted to 7.4 with NaOH), (2) 10 min with Ca^2+^-free Tyrode’s solution, (3) 25–30 min with the digestion solution consisting of Ca^2+^-free Tyrode’s solution, to which was added 0.03 mM CaCl_2_, 20 mM taurine, 0.1% Bovine Serum Albumin (BSA), and 73.7 U/mL type II collagenase (Worthington Biochemical Corporation, NJ, USA), and (4) 3 min with Kraft-Brühe (KB) solution (in mM: 100 K^+^-glutamate, 10 K^+^-aspartate, 25 KCl, 10 KH_2_PO_4_, 2 MgSO_4_, 20 taurine, 5 creatine, 0.5 EGTA, 5 HEPES, 20 glucose, 0.1% BSA, pH adjusted to 7.2 with KOH). Left atrium was then isolated, minced, and triturated until individual atrial myocytes were obtained. Freshly isolated myocytes underwent a Ca^2+^ readaptation, during which Ca^2+^ was progressively reintroduced in 5-min steps at 0.06, 0.12, 0.24, 0.6, and then 1 mM Ca^2+^. The cells were then stored at 4 °C until use, typically one to six hours later. Rod-shaped myocytes were selected for experiments.

### 4.4. Voltage Clamp Experiments

Aliquots of left atrial myocytes were placed in a perfusion chamber of an inverted microscope, maintained at 37 °C, and continuously perfused with oxygenated solutions at 2 mL/min. Cells were given 10 min to settle to the bottom of the chamber before the start of solution flow. Voltage-clamp current recordings were acquired with an Axopatch 200B patch-clamp amplifier and pCLAMP 10.2 software (Molecular Devices, Sunnyvale, CA, USA), using whole-cell configuration. Pipettes were pulled from borosilicate glass (World Precision Instruments, Sarasota, FL, USA) and had resistances between 2 and 4 MΩ when filled with the pipette solutions. Recordings were acquired at a sampling rate of 4 kHz. All currents are expressed as current densities (pA/pF). Voltages were not corrected for liquid junction potentials as they were calculated to be less than 4 mV. All experiments were carried out at 37 °C.

*NCX1 current (I_NCX_).* The protocol used to record I_NCX_ was adapted from Ozdemir et al. [55]. I_NCX_ was recorded in whole-cell configuration using pipettes filled with the following solution (in mM): 65 CsCl, 10.92 CaCl_2_, 20 EGTA, 10 HEPES, 0.5 MgATP, 20 TEA-Cl, and 20 NaCl (pH adjusted to 7.2 with CsOH), for an estimated free [Ca^2+^] of 150 nM. The voltage protocol consisted of a holding potential of −40 mV with a ramp from +80 to −120 mV over 2 s. Cells were perfused with a solution containing (in mM): 140 NaCl, 10 TEA-Cl, 12 HEPES, 0.5 MgCl_2_, 2 CaCl_2_, and 10 glucose (pH adjusted to 7.4 with NaOH). A MPRE8 Multitube Preheater (Cell MicroControls, Norfolk, VA, USA) was used to ensure rapid and local change of perfusion solutions. A baseline current was recorded in the following bath solution (in mM): 140 NaCl, 10 TEA-Cl, 12 HEPES, 0.5 MgCl_2_, 4 CaCl_2_, 1 BaCl_2_, 0.02 Nifedipine, and 0.005 Ryanodine (pH adjusted to 7.4 with NaOH). Then, the current was recorded again after adding 5 mM of nickel chloride (NiCl_2_) to obtain I_NCX_ as the nickel-sensitive current. High concentrations of internal Na^+^ and external Ca^2+^ were used to maximize electrophysiological gradients of NCX1 to generate currents of greater amplitude to compensate for the lower cellular capacitance of atrial myocytes compared to ventricular myocytes [56,57].

*I_CaL_.* Protocols used to record I_CaL_ were adapted from Bögeholz et al. [23]. Briefly, atrial myocytes were perfused with Tyrode’s solution containing 2 mM of CaCl_2_. Cells were voltage-clamped in whole-cell configuration using borosilicate glass pipettes filled with (in mM): 120 CsCl, 10 TEA-Cl, 10 NaCl, 20 HEPES, 5 MgATP, and 0.05 cAMP (pH adjusted to 7.2 with CsOH). When specified, BAPTA (10 mM) was added to the pipette solution to buffer intracellular Ca^2+^. From a holding potential of −80 mV, a 50 ms pre-pulse at −50 mV was applied followed by a series of 250 ms voltage steps ranging from −50 to +60 mV in 5–10 mV increments, at a frequency rate of 0.1 Hz. I_CaL_ was defined as the difference between the peak current and that measured at the end of the 250 ms test pulse. Protocols used to study I_CaL_ kinetics are provided in the Supplementary Materials.

### 4.5. Calcium Transients

Ca^2+^ transients from mouse atrial myocytes were recorded using adaptation of previously published protocols [50,58]. For these experiments, atrial myocytes were maintained in KB solution without EGTA at the end of digestion. Atrial cells were incubated for 30 min at room temperature (RT) with 5 µM of Fluo-4-AM (Molecular Probes, Eugene, OR, USA). Cells were then placed in the recording chamber of a Zeiss LSM710 (Zeiss, White Plains, NY, USA)confocal microscope and given 15 min to adhere to laminin-coated coverslips. Cells were maintained at 35 ± 1 °C, perfused with 1 mM of Ca^2+^ Tyrode’s solution for 10 min, and then stimulated for an additional 5 min before starting the recording session. Each recording consisted of 10 s of pacing at a frequency of 2 Hz using field stimulations, 10 s without pacing, and 5 s of local perfusion with Ca^2+^-free Tyrode’s solution containing caffeine (10 mM) via a rapid solution switcher (MPRE8 Multitube Preheater Cell MicroControls). The recording lasted a total of 40 s to allow complete decay of the caffeine-induced transient. Data were acquired at a sampling rate of 100 Hz. Fluo-4 was excited at 489 nm and emission was read at 505 nm. Data were extracted using Zen Black 2.0 software (Zeiss, White Plains, NY, USA) and was analyzed manually using Clampfit 10.7 software (Molecular Devices, Sunnyvale, CA, USA). 

Spontaneous systolic and diastolic Ca^2+^ releases were analyzed manually. The inclusion criterion for spontaneous systolic Ca^2+^ release was a spontaneous increase in fluorescence during the decay phase of a Ca^2+^ transient during stimulation at 2 Hz. Spontaneous diastolic Ca^2+^ releases were defined as transient increases in fluorescence, clearly distinguishable from noise level when cells were unstimulated. Each spontaneous event was confirmed by video visualization using Zen Black 2.0 software (Zeiss, White Plains, NY, USA).

### 4.6. qPCR

Total RNA extraction and qPCR were conducted using previously published protocols [51]. Total RNA was extracted with TRIzol Reagent™ (Ambion, Austin, TX, USA) using one left atrium per sample (n). Each RNA sample underwent a purification coupled with a DNase treatment using the Nucleospin RNA kit (Machery-Nagel, Düren, Germany), following the manufacturer’s instructions. Reverse transcription was achieved using the High-Capacity cDNA Reverse Transcription Kit (Applied Biosystems, Waltham, MA, USA). qPCR was performed using SYBR Select Master Mix (Applied Biosystems, Waltham, MA, USA) on a Quantstudio 3 system (Applied Biosystems, Waltham, MA, USA). Each sample was analyzed in duplicate and gene expression was normalized to the housekeeping gene *Hypoxanthine Guanine PhosphoribosylTransferase 1* (*Hprt1*). The relative expression was calculated using the 2^-∆∆Ct^ method. Murine gene-specific primers used for qPCR reactions are presented in Table S1.

The protocol used to isolate total RNA from mouse atria was also used on human samples. Atrial biopsies were collected from explanted hearts after transplantation and provided by the human tissue bank of the Montreal Heart Institute (project #2007-43). We quantified the mRNA expression of *SLC8A1* and *CACNA1C* in atrial samples from 5 men and 6 women with no history of cardiac diseases. Gene expression was normalized to the housekeeping gene *HPRT1*. Human gene-specific primers used for qPCR reactions are presented in Table S2. Only one replicate of each sample was analyzed due to the low cDNA content. 

### 4.7. Western Blot

Protocols used for the isolation of the total protein fractions and Western blot analysis were adapted from our previously published papers [51,59]. Briefly, each sample contained two left atria and was homogenized in ice-cold extraction buffer containing a mixture of protease inhibitors [59]. Triton was added to the homogenate and the samples were incubated on ice for 2 h. After a 10-min centrifugation at 10,000 g at 4 °C, the supernatant was collected. Protein concentrations were assessed by a standard Bradford assay. Proteins (30 µg) were loaded and electrophoretic separated in Stain-Free gels (TGX 7.5% Stain-Free; Bio-Rad Laboratories). Samples were not heated prior to loading. For Ca_V_1.2, proteins were transferred overnight at RT at low voltage (10 V) onto a polyvinylidene difluoride (PVDF) membrane. The membrane was fixed for 30 min at RT in the following buffer: 70% methanol, 29% acetic acid, and 1% glycerol. The membrane was then blocked in Tris-buffered saline Tween-20 solution (TBST) containing 5% non-fat dry milk for 4 h, at RT, and then incubated overnight at 4 °C with primary antibody (1:10, Neuromab 73–053 mouse anti-CaV1.2). For NCX1, the electrophoretic separation was followed by a 90-min transfer at 350 mA onto PVDF membrane at 4 °C. The membrane was blocked for 90 min with TBST containing 5% BSA at RT and then incubated overnight at 4 °C with mouse anti-NCX1 antibody (1:10,000, Swant R3F1). The lower sections of both Ca_V_1.2 and NCX1 membranes were cut, and independently blocked with TBST/5% non-fat dry milk and incubated with anti-GAPDH (1:25,000, Sigma mouse monoclonal G9295 antibody). Following three 5-min washes, all membranes were incubated in secondary antibodies (horseradish peroxidase-conjugated goat anti-mouse) in TBST/5% non-fat dry milk for 90 min at RT. After 3 additional TBST washes, bands of interest were detected using enhanced chemiluminescence reagents (ECL plus, ParkinElmer. Woodbridge, ON, Canada) and visualized in film-free chemiluminescence using ChemiDoc apparatus (Bio-Rad, Hercules, CA, USA). Densitometry analysis was performed using Bio-Rad Image Lab 6.1. The expressions of Ca_V_1.2 and NCX1 were normalized to their respective loading controls, GAPDH.

### 4.8. Statistical Analysis

Results are presented as mean ± standard error of the mean (SEM) unless specified otherwise. For patch-clamp and Ca^2+^ transient experiments, n indicates the number of cells and N represents the number of mice. The compared groups were tested for equality of variance. Unpaired two-tailed Student’s t-tests were used for numerical data, whereas the chi-squared test was used for categorical data such as EPS data and spontaneous Ca^2+^ releases. Statistical analysis was performed using GraphPad Prism 9.4 software (GraphPad Software, San Diego, CA, USA). *p*-values inferior to 0.05 were considered statistically significant.

## 5. Conclusions

This study showed that, as in humans, male mice have a greater vulnerability to AF. Moreover, we identified important sex differences in Ca^2+^ handling regulation associated with higher NCX1 expression and function and more frequent spontaneous Ca^2+^ releases in males. Together, these results suggest that males are more prone to developing AF due to increased triggered activity. Equally important, these sex differences were observed independently of risk factors such as aging and comorbidities.

## Data Availability

The data that support the findings of this study are available from the corresponding author on reasonable request.

## Supplementary Materials

The following supporting information can be downloaded at: https://www.mdpi.com/article/10.3390/ijms231810724/s1.
