# Supplementary material for: Higher Na+-Ca2+ Exchanger Function and Triggered Activity Contribute to Male Predisposition to Atrial Fibrillation
Source: Int J Mol Sci. 2022 Sep 14;23(18):10724. doi: 10.3390/ijms231810724 (PMC9501955; doi:10.3390/ijms231810724)
Supplement: Supplementary file 1 [file ijms-23-10724-s001.zip › ijms-1910746-supplementary.pdf]

This pdf includes:

- Supplemental methods
- Supplemental Figures 1 to 4
- Supplemental Tables 1 to 3

Supplemental methods:

I_{CaL} inactivation protocols. The voltage dependence of I_{CaL} steady-state inactivation was measured using a two-step voltage-clamp protocol consisting of a first 500-ms inactivating step at selected potentials (from -80 mV to 20 mV, in 5-10 mV increments), followed by a 250-ms test step to 0 mV. In addition, a 10-ms prepulse at -50 mV was added before the 250-ms test step to inactivate I_{Na}. The current amplitude at each first pulse potential was normalized to the maximal amplitude of the current ($I/I_{max)}$ and plotted as a function of the inactivating prepulse potential. Data were fitted to a Boltzmann equation: $I/I_{max} = 1/[1+I_{exp} ((V_m-V_{1/2})/ S_{1/2})]$, where $V_{1/2}$ represents the membrane potential (V_m) when 50% of the channel are inactivated and $S_{1/2}$ is the mid-point slope factor. Protocol for the recovery from inactivation of I_{CaL} consisted of a holding potential of -50 mV, two 250-ms test steps at 0 mV separated by a recovery step at -50 mV lasting from 10 ms to 2000 ms.

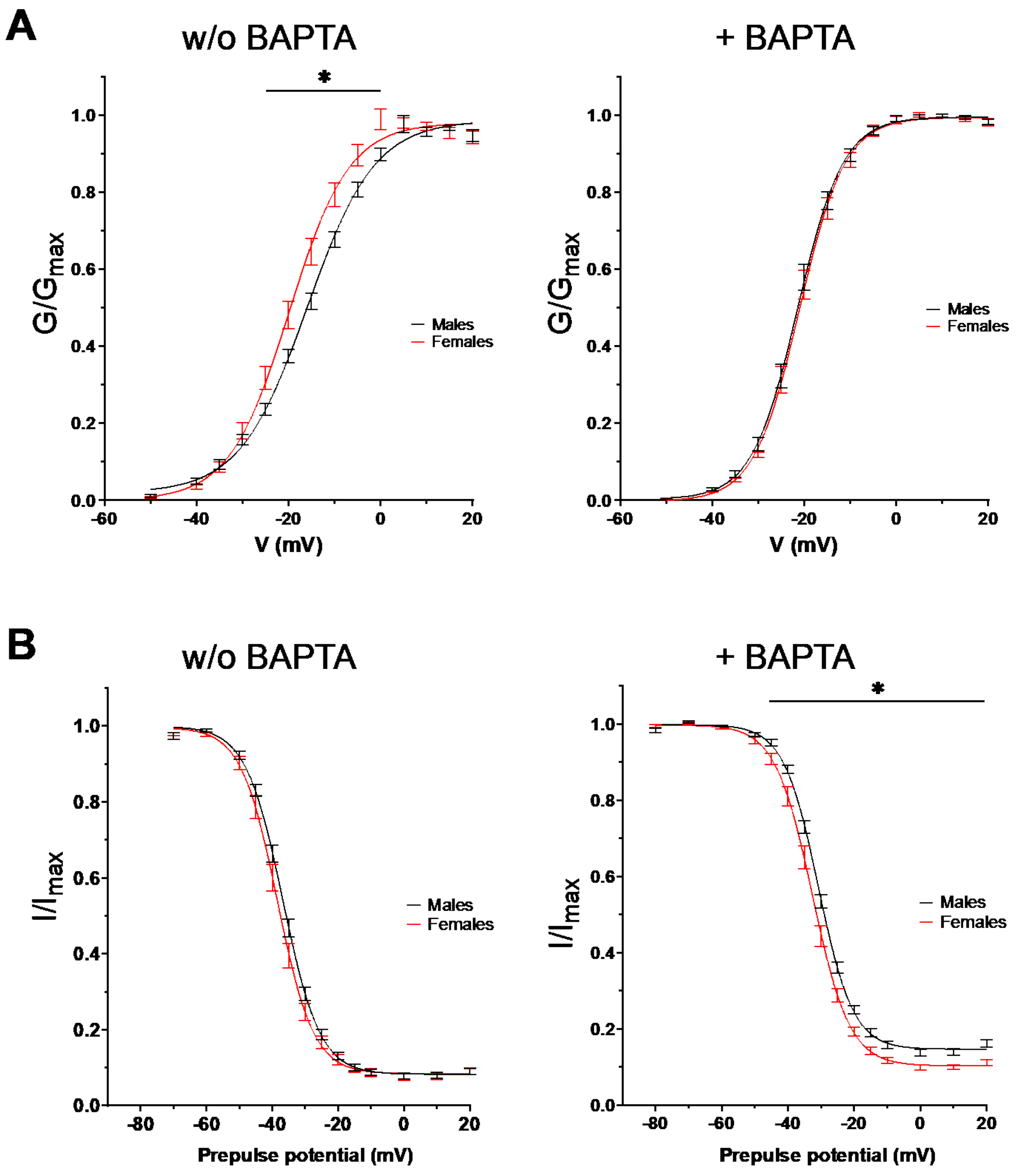

Supplemental Figure S1. Steady-state activation and inactivation of I_{CaL} in absence or presence of BAPTA, in male and female mouse atrial myocytes. A. Left. Steady-state activation curve for I_{CaL} shows a positive shift in males in absence of BAPTA and a slower activation slope in comparison to females. Right. There is no sex difference in the voltage dependence of the steady-state activation in presence of BAPTA. B. Steady-state inactivation of I_{CaL} current in male and female atrial myocytes without (left) and with BAPTA (right). Numerical values for I_{CaL} kinetics are provided in supplemental table S3.

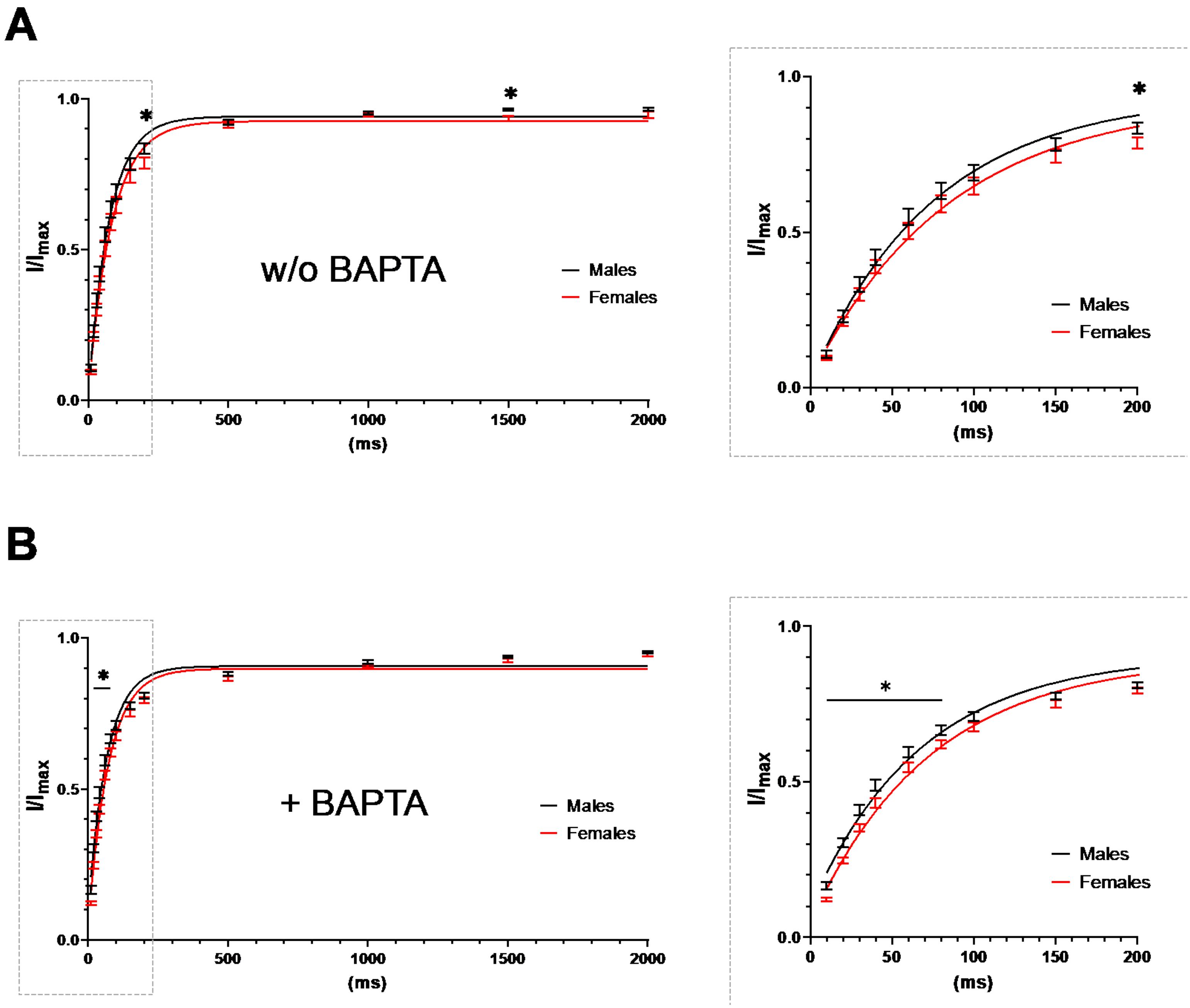

Supplemental Figure S2. Recovery from inactivation of I_{CaL} in absence (A) and presence (B) of BAPTA in male and female mouse atrial myocytes. Left panel shows mean values of relative current against interpulse interval for the entire time scale (10 to 2000 ms). Right panel presents the data over an expanded time scale (10 to 200 ms). Solid lines are best-fit single exponential functions. Rate constant for recovery from inactivation without BAPTA, at holding potential of -50 mV were 0.0132 ms^{-1} (95% CI: 0.0120 to 0.01462 ms^{-1}, n=23, N=3) for males and 0.0136 ms^{-1} (95% CI 0.0117 to 0.0130 ms^{-1}, n=13, N=2) for females. In presence of BAPTA, these values were 0.0148 ms^{-1} (95% CI: 0.0135 to 0.0162 ms^{-1}, n=24, N=3) for males and 0.0136 (95% CI 0.0127 to 0.0147 ms^{-1}, n=18, N=2) for females.

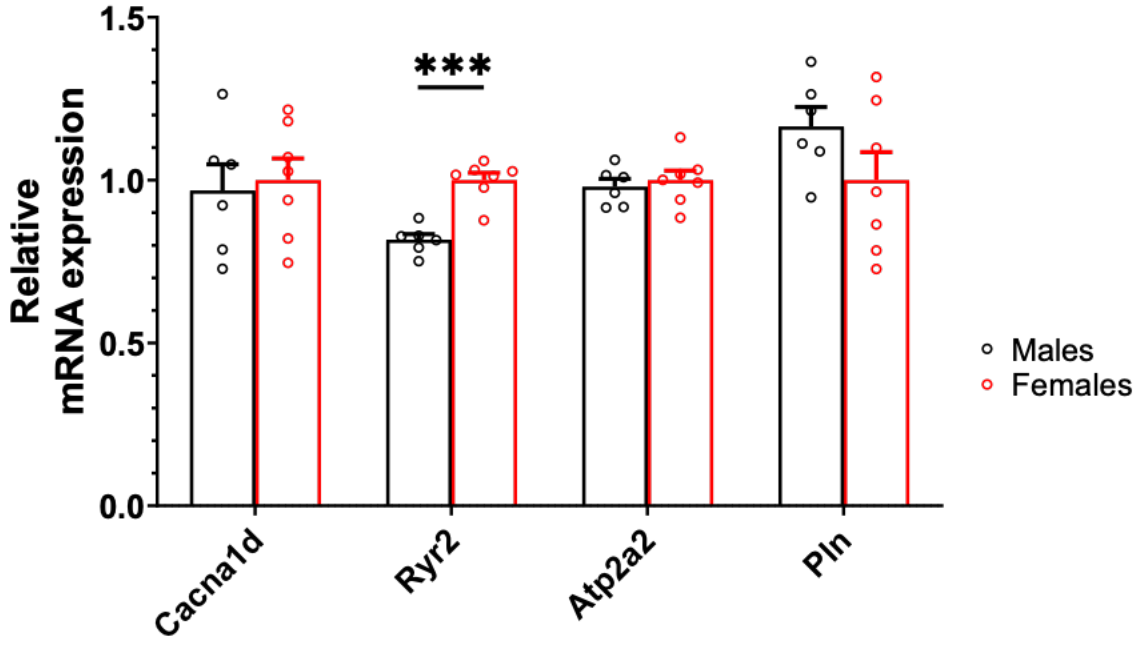

Supplemental Figure S3. Relative mRNA expression of Ca^{2+} handling proteins in mouse atria. Scatter plots displaying mRNA expression in left atria of male and female mice for the following genes: *Cacna1d* (coding for $Ca_V1.3$), *Ryr2* (coding for Ryanodine Receptor 2) *Atp2a2* (coding for SERCA2A) and *Pln* (coding for Phospholambam) (M: N = 6; F: N = 7). Sex differences were only seen in *Ryr2* expression (M: 0.82 ± 0.02, F: 1.00 ± 0.02, p = 0.00007).

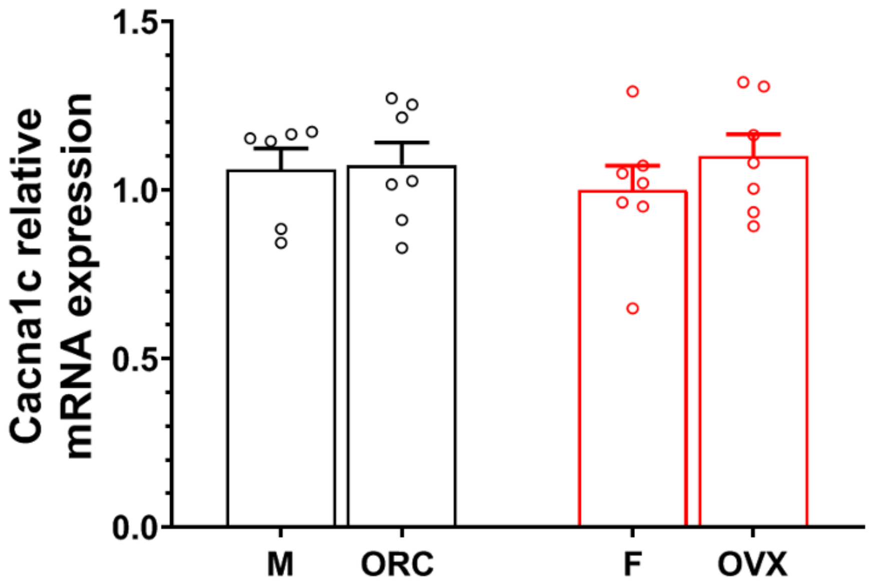

Supplemental Figure S4. Atrial *Cacna1c* gene expression is not affected by orchiectomy (ORC) and ovariectomy (OVX) in mice. Scatter plot shows no difference in the expression of *Cacna1c* in ORC and OVX mice compared to their respective controls (N = 6-7/ group).

Supplemental Table S1. Primer sequences used for qPCR analysis in mice

Target gene	Forward primer sequence	Reverse primer sequence
Hprt1	TGA ATC ACG TTT GTG TCA TTA GTG A	TTC AAC TTG CGC TCA TCT TAG G
Cacna1c	CGT TCT CAT CCT GCT CAA CAC C	GAG CTT CAG GAT CAT CTC CAC TG
Cacna1d	CTA CCG TTG CAC AGA TGA AGC C	TCA CGG ACC ACA GGA CTG TCA A
Slc8a1	AGA GGA GGA GAG GCG CAT TG	CGC TGA CAG TGA TGG CTT CG
Ryr2	CAT GAG AAT GCT GGC CTT GT	TAG CAG TAT CGC TGG AGG TT
Atp2a2	CTG GAG GTT AAT ACT GAG ATC GGC A	TCC AGA CTG CAA TGC AAA TGA G
Pln	TGC CCA GCT AAG CTC CCA TA	GCT GGC AAG TTC CTT TGG TC

Supplemental Table S2. Primer sequences used for qPCR analysis in humans

Target gene	Forward primer sequence	Reverse primer sequence
HPRT1	TGC TGA CCT GCT GGA TTA CA	TTT ATG TCC CCT GTT GAC TGGT
CACNA1C	GCG CCG CAG TCA AGT CTA AT	AGG AGC ATC TCT GCC GTG AA
SLC8A1	GTG TTC GTC GCA CTT GGA AC	TGC CCG TGA CGT TAC CTA TG

Supplemental Table S3. Activation and inactivation kinetics of I_{CaL} in absence and presence of BAPTA in left atrial myocytes of male and female mice

		Without BAPTA			With 10 mM BAPTA		
		Males	Females	*p-value*	Males	Females	*p-value*
Steady-state activation	$V_{1/2}$	**-15.8 ± 0.6**	**-19.1 ± 0.9**	**0.002***	-21.3 ± 0.6	-20.5 ± 0.7	0.40
	Slope	7.7 ± 0.5	6.6 ± 0.3	0.086	4.7 ± 0.3	4.5 ± 0.25	0.52
	n	22	21		26	25	
Steady-state inactivation	$V_{1/2}$	**-36.8 ± 0.5**	**-38.5 ± 0.8**	**0.05***	**-30.8 ± 0.4**	**-32.7 ± 0.7**	**0.02***
	Slope	5.4 ± 0.1	5.2 ± 0.2	0.23	5.2 ± 0.1	5.3 ± 0.1	0.47
	n	24	14		24	22	
Macroscopic inactivation (at -5 mV)	Tau	7.2 ± 0.3	6.5 ± 0.2	0.09	10.3 ± 0.4	10.0 ± 0.4	0.53
	R10	**0.29 ± 0.02**	**0.25 ± 0.01**	**0.05***	0.44 ± 0.01	0.42 ± 0.01	0.35
	R20	**0.14 ± 0.01**	**0.11 ± 0.01**	**0.005***	0.20 ± 0.01	0.19 ± 0.01	0.65
	R40	**0.060 ± 0.005**	**0.042 ± 0.003**	**0.002***	0.068 ± 0.003	0.068 ± 0.003	0.98
	n	**22**	**21**		**26**	**24**	
